# Simplifying Forehead and Temple Reconstruction: A Narrative Review

**DOI:** 10.3390/jcm12165399

**Published:** 2023-08-19

**Authors:** Pedro Redondo

**Affiliations:** Department of Dermatology, University Clinic of Navarra, 28027 Madrid, Spain; predondo@unav.es; Tel.: +34-948255400

**Keywords:** forehead, temple reconstruction, frontalis myocutaneous transposition flap, preauricular skin advancement flap, glabella, eyebrows

## Abstract

The forehead and temporal region are frequent areas of skin cancer development. After tumor removal, reconstruction must be performed, maintaining the frontal–temporal line of the scalp and symmetry of the eyebrows in an attempt to hide the scars within these marks or natural folds and wrinkles. Second wound healing and skin grafts generally do not produce an acceptable cosmetic result. When direct closure is not possible, the technique of choice is skin flaps. In the midfrontal line continuation of the glabella, there is a remnant of skin to be used as a donor area for local flaps; similarly, it occurs in the preauricular cheek, which can move toward the temple. In addition to the classic advancement and rotation flaps, the frontalis myocutaneous transposition flap is an excellent technique for closing defects which are wider than higher on the forehead. Its design is very versatile and can be performed between the two pupil lines at different heights depending on the location of the defect. On the other hand, the preauricular skin advancement flap with an infralobular Burow’s triangle is also an excellent option for reconstructing tumors in the temporal area.

## 1. Introduction

In the reconstruction of the forehead and temple, we find at least five different anatomical subunits: the glabella, eyebrows, median forehead, lateral forehead and temple. It is also advisable to divide these last three areas into superior and inferior, with the superior region limiting with the implantation line of the scalp and the inferior region with the eyebrows or with a concave line between the outer canthus and the inferior portion of the tragus [1].

Several goals must be considered in reconstructive surgery of the forehead and temple: preservation of motor function (the temporalis branch of facial nerve); maintenance of the normal position and symmetry of the brow and the frontotemporal hairline; and optimal scar camouflage through the placement of scars adjacent to the hairline and brow and in relaxed skin tension lines whenever possible, which are generally horizontal and parallel in the median and lateral forehead and curvilinear in the temple [2]. In the glabella and at the beginning of its prolongation toward the scalp, wrinkles may be vertical or curvilinear. Logically, the wrinkles and folds become more prominent with age and are hardly noticeable in younger patients [3]. In the continuation of the outer canthus toward the tragus, there are normally some marked wrinkles, also popularly called “crow’s feet”, where some scars may almost always be successfully hidden.

Split- and full-thickness skin grafts do not usually produce satisfactory results on the forehead and temple. These grafts are usually patch-like on an otherwise ungrafted face, and their edges are their major shortcoming unless hidden along natural lines. Skin grafts provide a poor match in thickness and color, and at best, should be considered a temporary measure in patients with complex reconstructions or very aggressive tumors with a high risk of recurrence. For these reasons, from a cosmetic point of view, the use of grafts in a facial area as visible as the forehead or temple is not the first choice [4,5]. Skin grafts cannot be used in deep defects exposing the outer table. When there is insufficient skin to close the defect, the excised triangles can be used as Burow’s grafts because these will have a texture and color similar to those of the excised skin.

Without a doubt, the best reconstructive solution in dermatologic surgery is direct closure using a spindle, which should always be tested on both the forehead and temple [6,7,8]. By means of the design of lateral triangles, direct closure may be a straight line, a curved line or with an italic “S” shape, depending on the tension lines of the subunit in question. Pinching the edges will provide some orientation of the viability of direct closure and whether this maneuver leads to excessive traction/elevation of the eyebrow. Although due to the gravitational trend what normally goes up finally comes down, we must avoid this traction in young patients in whom the asymmetry may be permanent or very long-lasting. Primary closure is not considered amenable for large defects.

**Figure 1:**
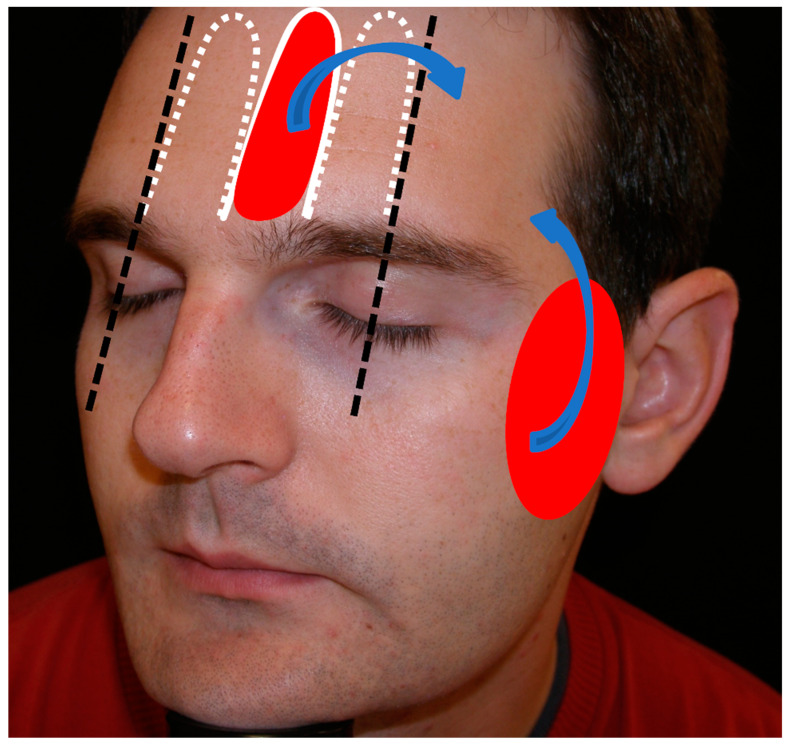
In the midfrontal line continuation of the glabella, there is a remnant of skin to be used as the donor area for local flaps; similarly, it occurs in the preauricular cheek, which can move toward the temple. The frontalis myocutaneous transposition flap design is very versatile and can be performed between the two pupil lines at different heights, depending on the location of the defect.

After surgery, larger forehead and temple defects are best repaired with flaps [9,10]. The preauricular cheek and median forehead are excellent reservoirs of skin, which are larger with increasing patient age, and they have a greater laxity than other anatomical areas, with a rich vascular network that allows for a large superficial area to be lifted with a low risk of necrosis (Figure 1).

Cheek advancement flaps have the advantage of relative mobility and elasticity of the skin and soft tissue of the cheek.

The general approach of many surgeons to defects with the above characteristics is a total skin graft or closure via secondary intention healing, because these techniques are considered to be safer, less costly and more comfortable for the patient. I do not agree [11]; and although the time of surgery using local flaps could be minimally longer than other procedures, the advantages are manyfold: (i) reconstruction of the forehead and temple can be performed with local anesthesia in patients who are not excessively anxious, otherwise, general anesthesia can be used; (ii) the stitches are removed at 6 days, and they do not need any special care; (iii) second healing of a similar defect requires at least 4 to 6 weeks of wound care and follow-up, and can produce some good results, especially in the temple region, but very often, the skin is depressed, discolored and filled with telangiectasia; (iv) a graft requires immediate follow-up, which often prolongs hospital stay and subsequent wound care; (v) skin grafts also require an additional surgical procedure at a distant site; (vi) and the final cosmetic outcome, which is essential even in the oncologic surgery setting, is much more satisfactory with advancement and transposition flaps of the forehead, temple and lateral cheek than with these other procedures.

## 2. Anatomy

The forehead is limited superiorly and inferiorly with the forehead implantation line of the scalp and eyebrows. Laterally, it contacts with the temple or temple region, which in turn extends from the temple implantation line of the scalp to the continuation of the outer canthus through a concave line which begins in the inferior portion of the tragus. Therefore, we may speak of a median line or median forehead, which corresponds to the interciliary region or the prolongation of the glabella, and the lateral forehead, which corresponds to the prolongation of the eyebrow and upward, and finally the temple or temple region, which begins medially at the tail of the eyebrow. The glabella is limited inferiorly by the nasal bridge, laterally by the root of the eyebrows and above by the median line, thus acquiring a shape similar to an equilateral triangle [1].

The vascularization of the forehead is very good and is based principally on two axial arteries on each side: the supraorbital artery and the supratrochlear artery. The forehead flap is a workhorse for reconstruction of large full-thickness nasal defects. It is characterized by its dependability, consistent anatomy and excellent texture match. For years, 180° transposition paramedian forehead flaps based on supratrochlear or supraorbital supply have been performed as part of a two-stage surgical procedure. Apart from these two arteries, from downwards to upwards, a large number of branches and small parallel vessels emerge, which allow for the design of paramedian forehead flaps at random without the need to search for them with Doppler ultrasound or include them in the design of the axial flap. Laterally, in the temple, the superficial temporal artery is found, i.e., the final part of the facial artery with its distal ramifications.

The sensitivity of the forehead depends on the supraorbital and supratrochlear nerves, especially the former, which emerge from the hole of the same name over the supraorbital notch. To anesthetize the forehead, nerve-block anesthesia on the infraorbital nerve can be used. To increase the desensitized area, tumescent anesthesia in a fan pattern may be deeply infiltrated one centimeter above the eyebrow, including the interciliary region. Regarding the minor secondary effects of surgery in this location, sensitive nerves may be traumatized or transected during dissection, and prolonged temporary anesthesia of the forehead and scalp may be present for 3 to 6 months postoperatively.

From the motor function point of view, enervation of the paraciliary and forehead musculature depends on the temporal branches of the facial nerve [12]. These branches become superficial after the parotid gland and may be damaged in the middle of the Pitanguy line from tragus to the lateral brow. The nerve traverses from a deeper subsuperficial musculoaponeurotic system (SMAS) plane to a more superficial sub-SMAS plane. The frontal branch of the facial nerve continues coursing superiorly before traversing from a sub-SMAS plane to a supra-SMAS plane both above the zygomatic arch and posterior to Pitanguy’s line. In this area, the temporal branch is more superficial and could be damaged if an incision is made or deep tissue detachment occurs. This is the most critical point as far as neurovascular damage is concerned regarding reconstruction of the forehead and temple.

In the following sections, we will review the reconstructive technique of choice according to the anatomical sub-unit.

## 3. Median and Lateral Forehead Reconstruction

### 3.1. Advancement Flaps

Unilateral U-plasty and bilateral H-plasty are the most frequently used advancement flaps for forehead defects, with a quadrangular shape or those which are higher than narrower, but they are not a good option in patients with horizontally large wounds [13]. The defect is extirpated with a geometric form—square or rectangular—and the incisions are horizontal, parallel to the implantation line of the scalp and eyebrows. Detachment must be made on the deep subcutaneous plane, and it is normal for there to be blood vessels of moderate caliber (supertrochlear and supraorbital arteries), which are sectioned transversely. In the corners, two or four small Burow’s triangles may be drawn, depending on whether the advancement is uni- or bi-lateral [14]. When we use this reconstructive option in the lateral forehead, it must be kept in mind that the advancement proceeding from the temple or temple region must be more curvilinear than straight, and that the skin on this side is less displaceable than that from the median forehead, in part due to the underlying convexity. Another option is the A–T advancement flap, which is also very versatile for the forehead region, especially for vertically positioned triangular defects.

### 3.2. Rotation Flaps

Simple rotation flaps (with a small Burrow’s triangle at the tail of the flap) are the most common for triangular horizontally positioned triangular defects. In the proximity to the implantation line of the scalp, a double-rotation O–Z flap may be used. The superior part can be designed just on the edge of the implantation and the inferior part on the hairless forehead, depending on the size and location of the defect. Large oval defects on the lateral forehead, which lie close to the eyebrows and implantation line of the scalp, may be reconstructed using a variant of the same deign. In this case, displacement of the median forehead is achieved by rotating the tissue using an incision in the implantation line of the forehead, while the advancement/rotation of the temple is achieved after drawing and using a flap parallel to the eyebrow [15,16,17].

### 3.3. Transposition Flaps

In my opinion, the flap of choice is the frontalis myocutaneous transposition flap (FMTF) [18]. The greatest reservoir of skin on the forehead is in the median line, in the continuation of the glabella. The principle of the FMTF consists of its thickness, containing part of the frontalis muscle or its fascia, which ensures vascular supply and allows for a long and narrow design that makes the primary closure of the donor site possible. The forehead has rich vascularization with an extensive anastomotic network that allows for the use of axial pattern flaps without the named arteries. Various designs can be implemented to achieve eyebrow and hairline symmetry and carefully place suture lines within relaxed tension lines when possible.

**Figure 2:**
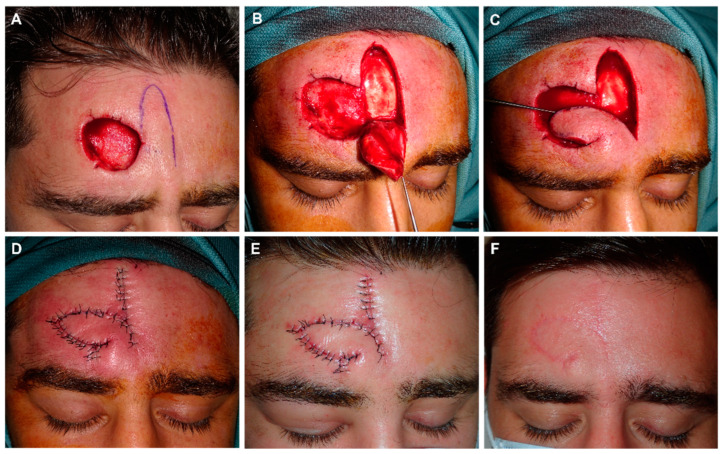
(**A**) Dermatofibrosarcoma protuberans on the frontal midline. Final defect. Design of a frontalis myocutaneous transposition flap. The individual forehead is carefully explored in each patient by laterally pinching the possible donor sites with fingers, and checking that their primary closure is possible. (**B**) Flap carved on the submuscular plane. (**C**) Transposition movement of the finger-like flap toward the defect. (**D**) Direct closure of the donor zone with clamps. Excision of a small Burow triangle associated with de-epithelialization of the turning point to prevent a bulge fold. With this maneuver, the bulge is reduced without damaging the pedicle. Immediate result. (**E**) Appearance after 24 h. (**F**) Appearance two months after surgery.

This flap allows for the reconstruction of oval or rectangular defects of the median or lateral forehead, which are wider than higher, and in which bilateral advancement may be very forced. The design is made randomly, with the shape of an axial finger-shaped flap where the supratrochlear and supraorbital arteries do not necessarily need to be included. In my opinion, the external limit for the design must be the pupillary lines, such that between the two lines, digits can be designed toward the superior forehead with the necessary length to close the adjacent defect (Figure 1). The flap is designed by prolonging the edge of the defect at a maximum angle of 90°. Ideally, the defects should be in the inferior portion of the forehead so that the design of the flap does not extend into the scalp (Figure 2). 

**Figure 3:**
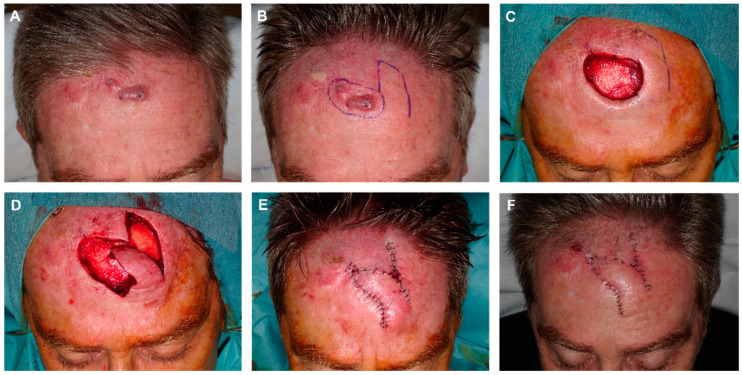
(**A**) Recurrent squamous cell carcinoma adjacent to the frontal hairline. (**B**) Excision margins for Mohs surgery and design of the reconstruction. (**C**) Final defect. (**D**) FMTF movement. (**E**) Immediate result after excision of the Burow triangle on the base of the defect. (**F**) Appearance 8 days later.

When the defects are partly in the superior portion of the forehead and partly in the implantation line/scalp, this design is very satisfactory, as when prolonging the design upward, part of the tissue displaced is hairy and the implantation line can be reconstructed [19] (Figure 3).

**Figure 4:**
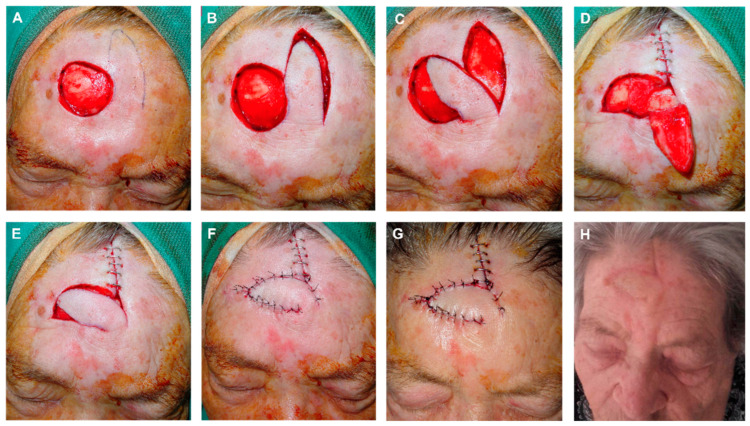
(**A**) Final defect with free margins down to the periosteum. Design of the finger-shaped transposition flap. (**B**,**C**) Flap sculpted on a deep plane, lifted and displaced. (**D**) Donor area sutured with clamps. Reduction in the defect height by a subcutaneous guitar-string suture. These vertical dermal–subcutaneous sutures begin at the depth of the wound and roll toward the surface, reenter the opposite side of the wound in the dermis and roll deep, thus creating uniform tension across the wound and significantly decreasing the size of the defect. (**E**) Flap pulled into place. De-epithelialization of the turning point to prevent a bulge fold; the epidermis and superficial dermis are removed to avoid this fold, but the rest of the layers maintained to ensure vascular supply of the FMTF. (**F**) Immediate result after closure with 4/0 polyglactin and 6/0 silk sutures. (**G**) Appearance 24 h after surgery. (**H**) Appearance one month after surgery.

In the design of the FMTF, some caveats are worthy of mention. First, the primary closure of the donor site determines the flap’s maximum width, so each patient should be carefully examined to ensure that there is a sufficient quantity of remaining tissue by pinching the skin. Second, the increased vascular supply by the muscle allows for a narrower base. Third, a guitar-string suture could be performed to reduce the superior-to- inferior surgical defect distance and improve the flap matching (Figure 4).

Finally, the skin fold caused by the 90° transposition movement can be superficially removed at the same surgical stage.

The FMTF permits a random design and a long and narrow shape that allows for the primary closure of the donor site and a one-stage reconstruction of large forehead defects.

## 4. Temple Reconstruction

Anatomically, the temple and lateral forehead are part of an embryonic fusion plane, where tumors may develop with a high degree of invasion and infiltration. Although most keratinocytic carcinomas in the temple are well defined, patients may present with aggressive or neglected tumors exhibiting extensive invasion, and the preferred treatment is Mohs surgery.

**Figure 5:**
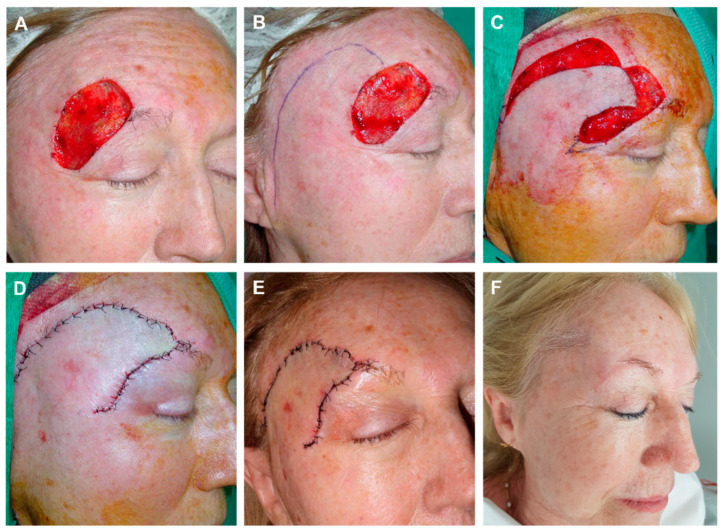
(**A**) Basal cell carcinoma on the temple and lateral eyebrow. Final defect. (**B**) Design of the rotation flap with minimal transposition. (**C**) Flap carved on the subcutaneous plane and partially rotated into place. (**D**) Immediate result after layered closure. (**E**) Appearance at 24 h. (**F**) Final result 6 months after surgery.

In this anatomic subunit, direct closure is often possible following the curvilinear tension lines or wrinkles parallel to the continuation of the tail of the eyebrow. Furthermore, we can hide the incisions transversally to these wrinkles by directing the vertex of the closure toward the outer canthus. Thus, in the temple, direct V–Y closure is a magnificent choice, drawing the scars in the “crow’s feet” of the prolongation of the outer canthus and in the hairy areas of the temple implantation of the scalp. This will depend on the size and traction that it produces on the tail of the eyebrow. In general, the greater the size, the better the indication for transversal closure. The temple is a region where second intention healing may be considered a reasonable functional and aesthetic alternative, although proximity to the eyebrow must be considered because scar contraction may result in brow elevation. In general, flaps in the temple region are more complex to perform. They displace less tissue due to the underlying convexity and may be less viable as they are less well-vascularized, as the plane of detachment must be more superficial to avoid damaging the facial nerve [20,21,22] (Figure 5).

### 4.1. Rotation Flaps

**Figure 6:**
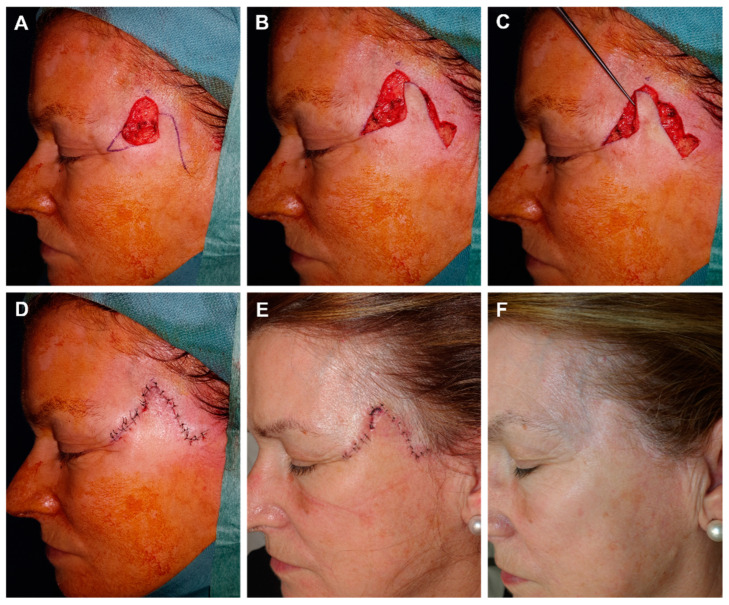
(**A**) Final defect after surgery of basal cell carcinoma in the temporal region. Design of an advancement flap with minimal rotation from the temporal area and preauricular Burow’s triangle. (**B**) Triangulation of the defect toward the outer canthus to reduce its size. (**C**) The flap displaced with the assistance of a dissecting hook covers a large part of the defect. (**D**) Immediate result without any tension. (**E**) Aspect 7 days later. (**F**) Appearance one year after surgery with an excellent aesthetic result.

Rotation flaps are a good choice for round or oval defects near the scalp (Figure 6).

The design of the flap, as usual, hides the scar in the implantation line of the temple. With this technique, we must avoid advancing the line of hair implantation and place more hair in the temple region. The nearer the incision and the detachment to the implantation line, the deeper it can be. This also makes it safer, as it is distant from the temporal branch of the facial nerve which is increasingly medial [23].

### 4.2. Advancement Flaps

**Figure 7:**
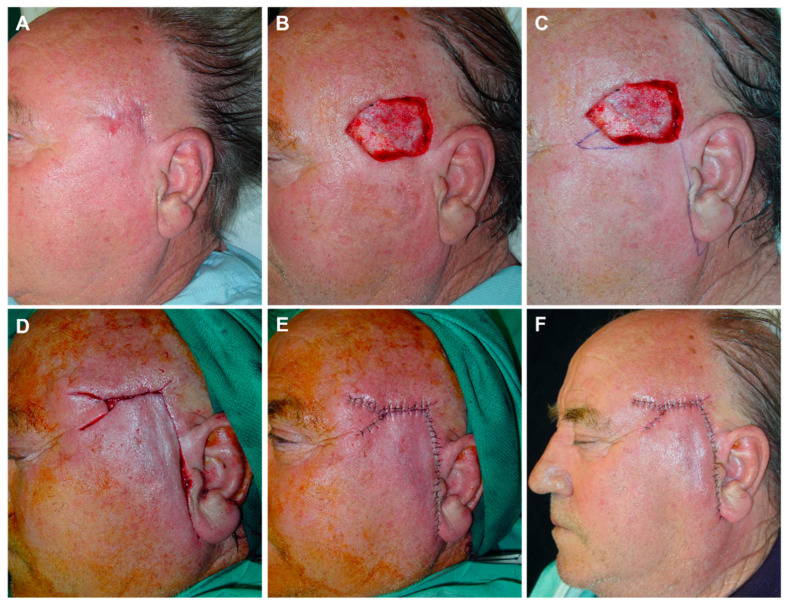
(**A**) A 75-year-old man with temporal basal cell carcinoma. (**B**) Final defect. (**C**) Design of a preauricular advancement flap with an infra-lobular Burow’s triangle. The most medial portion is prepared for an M-plasty to reduce the size of the scar and hide the incisions in the outer canthus and brow line. Flap lifted on the subcutaneous plane. (**D**,**E**) Immediate result after layered closure with 4/0 polyglactin and 6/0 silk. (**F**) Appearance after one week at suture removal, with most of the scars hidden within the natural folds.

In the preauricular region and the cheek, a great reservoir of skin exists which can be used to reconstruct defects of the temple (Figure 1). The advancement flap of preauricular skin and from the cheek is begun by making an incision from one of the edges of the defect toward the preauricular line, generally until the lobe is reached. There, Burow’s triangle is drawn, which can be hidden under the lobe. Posteriorly, the subcutaneous plane is detached, and the skin is displaced upward. This is a safe and viable plane, as all of the preauricular skin and the skin from the cheek have very good vascularization (Figure 7).

In my opinion, this flap is the first choice in the reconstruction of the temple. The flap is raised at the subcutaneous level, and attention should be paid to the course of the temporal branch of the facial nerve to avoid injury. This branch supplies motor innervation to the frontalis muscle and upper orbicularis oculi muscle, as well as the anterior and superior auricular muscles. It may be projected on the skin along a path drawn from a point 0.5 cm below the tragus to a point 1.5 cm above the lateral eyebrow. Sectioning of the temporal nerve results in an inability to wrinkle the forehead and raise the eyebrow.

The hair on the temple and the sideburn keeps the implantation line intact. The dog-ear that remains on the outer canthus after advancing and slightly rotating the flap can be excised and sutured, following the physiologic fold of the eyelids around the crow’s-feet area. Thus, all the incisions for the flap are within relaxed skin tension lines and are camouflaged. Meticulous hemostasis using atraumatic pinpoint electrocoagulation of the bleeding vessels should be performed before the flap is sutured.

**Figure 8:**
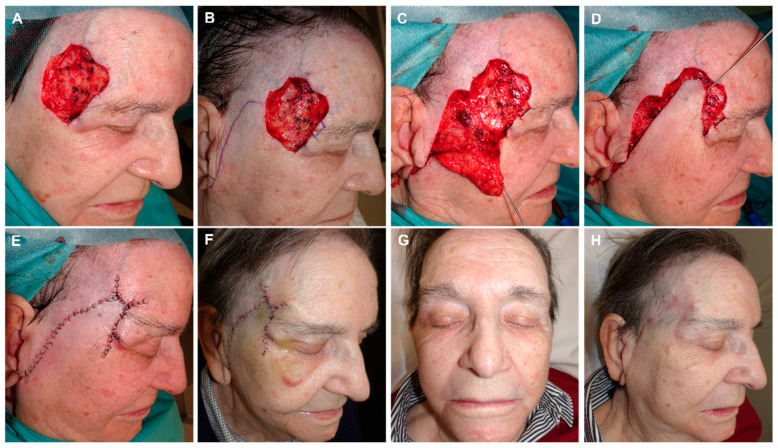
(**A**) Basal cell carcinoma on the temple. Initial defect with a positive lateral margin after 3D histology study. (**B**) Margin demarcated for extension and closure design using an advancement rotation flap from preauricular skin with an infra-lobular Burow’s triangle. (**C**) Flap lifted on the subcutaneous plane, (**D**) and pulled into place with a dissecting hook. (**E**) Design of three Burow’s triangles to reduce the size of the defect. Immediate result. (**F**) Appearance one week later. (**G**,**H**) Final aspect (front and lateral) 4 months after surgery.

In summary, advancing and slightly rotating the preauricular skin of the cheek, hiding Burow’s triangles in the supra- and infra-auricular area, is an excellent therapeutic option in the case of large defects located on the lateral forehead and temple (Figure 8).

The size of the defect to be covered may be reduced by designing Burow’s triangles on some of the edges which are directly closed, hiding the scars in the continuation of the external cantus (crow’s feet) or in the temple implantation line of the scalp.

Large reserves of skin are present, the cosmetic result is highly satisfactory and the only surgical risk is lesion of the temporal branch of the facial nerve. Even in thin individuals, there is usually sufficient subcutaneous tissue to allow for a subcutaneous dissection plane, preserving the subdermal plexus and flap vascularity.

**Figure 9:**
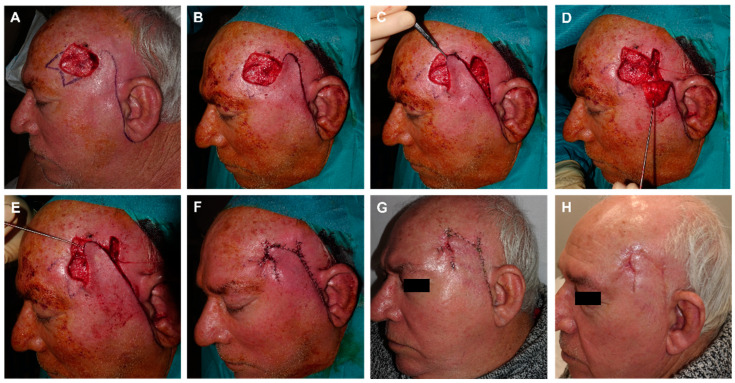
(**A**) Final defect after surgery of basal cell carcinoma on the left temple. Design of a preauricular advancement flap with an infra-lobular Burow’s triangle plus a small transposition flap on its distal end. (**B**) Flap lifted on the subcutaneous plane, (**C**) and pulled into place with a dissecting hook. (**D**,**E**) Guitar-string suture to reduce the width of the defect and donor site. (**F**) Immediate result after layered sutures with 4/0 polyglactin and 6/0 silk. (**G**,**H**) Appearance 1 and 10 days later, respectively.

In some cases, in the design of the flap, the movement of advancement or rotation of the skin can be combined with a degree of transposition, which can facilitate closure (Figure 9).

This is particularly useful for defects which may be more displaced toward the lateral forehead. Here, the movement of the temporal line of implantation of the scalp must be closely assessed, especially in women, but also as many older men have marked frontotemporal alopecia. 

Another option is the V–Y island advancement flap; although this flap can be great for horizontally positioned defects, it often leaves more marked scars with a poor cosmetic outcome.

### 4.3. Transposition Flaps

**Figure 10:**
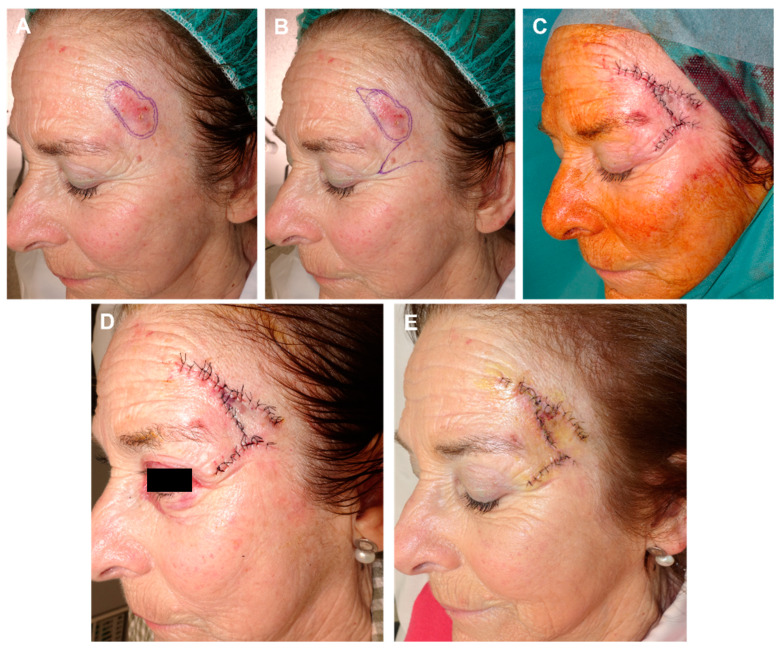
(**A**) Basal cell carcinoma on the left temple. (**B**) Design of a transposition flap from the temple skin, hiding one of the incisions over one of the crow’s feet. (**C**) Immediate result after suturing with 6/0 silk. (**D**) Appearance 24 hours later. (**E**) Final result after 6 days.

Defects of the temple located between the tail of the eyebrow and the implantation line of the scalp can be closed using the Limberg or Dufourmentel variant geometric transposition flap. The donor zone of a rhomboidal shape can be drawn in the “crow’s feet” prolongation of the outer canthus, in such a way that the direct closure is perfectly camouflaged and reduces excess tissue at this location, producing a lifting effect (Figure 10).

Other surgical options previously reported for repairing a bone-exposed forehead or temple defects include various random or pedicled local flaps, bilateral symmetric transposition flaps, subgaleal–subperiosteal flaps with split-thickness skin grafts, dermal matrices, tissue expansion or microvascular free tissue transfers.

## 5. Eyebrows Reconstruction

**Figure 11:**
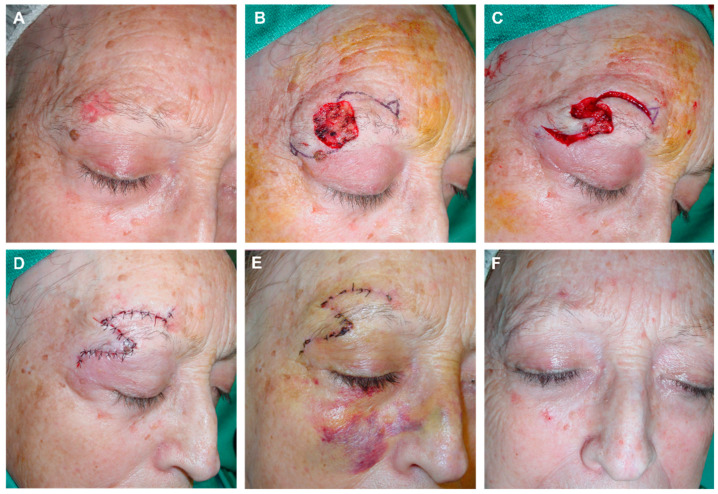
(**A**) Squamous cell carcinoma on the right eyebrow. (**B**) Final defect and design of an O–Z rotation flap with two small Burow’s triangles at both ends. (**C**) Flaps sculpted in a subcutaneous plane. (**D**) Immediate result after suturing in layers. (**E**) Appearance 7 days after surgery, at suture removal. (**F**) Appearance one and a half months after surgery.

For the reconstruction of eyebrows, there are two basic principles: maintaining symmetry and reducing the loss of hair follicles to a minimum [24,25,26]. Advancement flaps are usually the first choice (bilateral advancement flap or O to T advancement flap). Rotation flaps can be employed in eyebrow reconstruction with good cosmetic outcomes. Rotation flaps have utility with large defects that exceed the vertical height of the eyebrow (Figure 11).

## 6. Glabella Reconstruction

**Figure 12:**
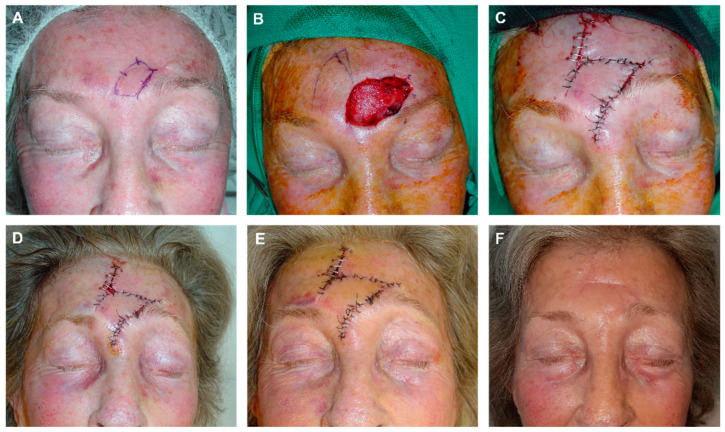
(**A**) Recurrent basal cell carcinoma in the interciliary area. (**B**) Final defect, which includes the frontal muscle and design of a transposition flap of the midline forehead. (**C**) Immediate result after layered suture. Excision of Burow’s triangle in the glabella and direct closure of the donor area with clamps. (**D**) Appearance 1 and (**E**) 8 days later. (**F**) Final result 6 months later.

**Figure 13:**
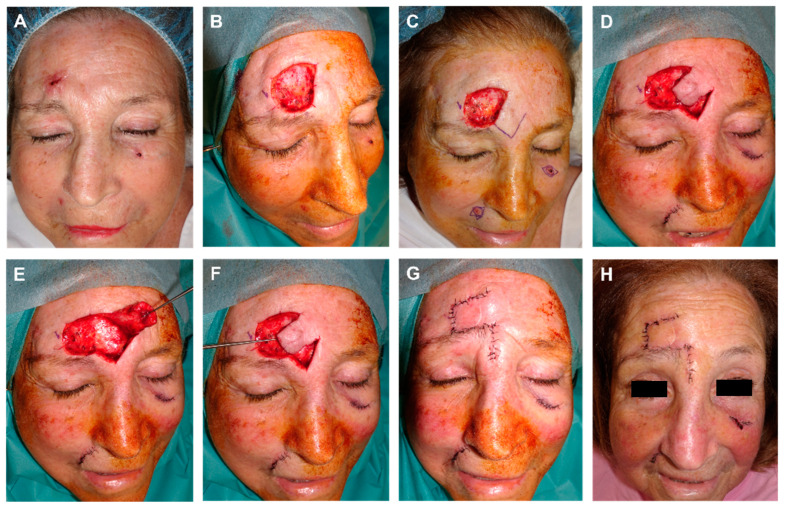
(**A**) Basal cell carcinoma on the forehead. (**B**) Final defect after 3D histology study. (**C**) Design of a transposition Limberg-like flap from the glabella. (**D**–**F**) Flap lifted on the submuscular plane, displaced with the a dissecting hook to cover the defect. (**G**) Immediate result after layered suture. (**H**) Appearance 8 days after surgery.

In the reconstruction of the glabella, we must follow expression lines or wrinkles which may be very variable—horizontal, vertical or curvilinear—in each individual. If they are not evident with the patient at rest, they should be marked by the patients forcing their expression. Furthermore, all attempts should be made to maintain the separation between the eyebrows, avoiding any loss of separation in that area. Direct closure is indicated for small defects. When these are larger, rotation flaps from the glabella itself or local transposition flaps are preferred, designed with a superior or inferior pedicle to avoid asymmetry or union of the eyebrows (Figure 12 and Figure 13) [9,10].

Another option to avoid the union of the eyebrows is the transposition flap from the nasal bridge, a transposition flap of 90° which minimally reduces the interciliary region with interposing tissue, generally in combination with a bilateral advancement of the forehead [27].

In summary, reconstruction of the forehead and temple is usually straightforward because of the availability of tissue, multiple options to close the defect, and numerous junction and skin tension lines in which to hide the surgical scars. In the case of a geometric defect, it is almost always possible to draw together the surrounding skin by directly making sutures after the design and excision of Burow’s triangles. If a flap is needed, tissue reservoirs of the median forehead and lateral cheek skin may be used to replace resected tissue with similar tissue, thereby restoring facial contours without distorting the surrounding functional and esthetic structures.

## Data Availability

Supporting data can be provided by contacting with the corresponding author.
